# Registration Difficulties.—II

**Published:** 1908-11-14

**Authors:** 


### Nursing Administration.

### REGISTRATION DIFFICULTIES.—II.

In order to give full weight to the objections urged against regulating the admission of nurses to the profession by examination, it must be borne in mind that qualifying examinations inevitably tend to stiffen up in natural response to the zeal of examiners, and their desire to raise the standard. Certain well-known societies started originally with examinations calculated to ensure a pass to every candidate who could read and write. And passing from the stage at which they conferred a high-sounding diploma on ignorance, they attained in course of years to the dignity of confounding simple-minded, though otherwise well qualified, examinees to whom scientific terminology proved a stumbling block. There is certainly some danger that the Central Midwives Board may eventually discourage altogether, by the severity of its examinations, the class of village midwives urgently required, if the provisions of the Midwives Bill are to take effect on the class for whose protection it was passed. And no one can doubt that great pressure would be put on any General Nursing Council to screw up the standard of theoretical knowledge to a point entirely beyond the reach of those slow wits who nevertheless often do work of incalculable value in the sick-room.

Passing from the consideration of this danger to the second chief objection commonly urged against the theory of registration for nurses, it is found that superintendents with a wide knowledge of the nursing world cherish grave distrust as to the effect of bestowing a life qualification on a particular section of nurses by the State. It is idle to contend that this measure is being promoted in favour of the whole body of nurses. It will affect merely the nurses trained in institutions recognised by the mysterious body known as the General Nursing Council, and of these only such as offer themselves for examination, and of these only such as can pass, and of these again only such as keep their names annually renewed. At every stage there will be a wastage, and in the aggregate it is doubtful whether as many as half the trained nurses of the country will have their names on the register. Of this more must be said under the head of practical objections to the registration of nurses. For the moment it is desired to concentrate attention on the effect on these registered nurses of the life qualification conferred on them by the State registration. We hear too much about the few black sheep who are to be excluded from the fold, and too little about the group who are safely folded in the pen of registration. What effect will their registration have upon the public and upon themselves? And first as regards the public. In certain callings registration is undoubtedly a protection to the public. The doctor who had been thoroughly taught his business is capable of setting a broken leg, or prescribing for ordinary ailments, even if for many years he has lived an irregular life and is absolutely indifferent to his patient's welfare. He may be a bad man and yet an able practitioner. In the same way a plumber who beats his wife and neglects his children may understand to a nicety the intricacies of drainage which earned him registration. In both these cases registration is a protection to the public, because it guarantees the possession of the vital qualifications essential to the functions of the medical man and the plumber. But registration does not in the least guarantee the possession by the nurse of the qualities essential to her profession. These qualities are so subtle and so quickly lost that nothing but testimonials from those who have watched her work, and these of recent date, are of any avail in guaranteeing them. What gradations from good to bad do not nurses pass through in ten years from the date of their training? How many causes are there which sap the efficiency of the nurse, and her suitability for the unlimited confidence to be reposed on her in the sick-room? Can it be pretended that the letters R.N. are going to protect the public from the faults most conspicuously complained of in trained nurses to-day? And what effect can registration have on the narrow group of registered nurses themselves? They consider the question, as any body of professional people is entitled and even bound to do, from the point of view of employment. Will it increase fees? Will it render employment more stable? Will it reduce competition? The expectation that it will do all these three things is answerable for much of the support which is being extended by private nurses to the proposals for legislation. We fear that a speedy disillusionment will await those who are cherishing golden visions on the strength of their ability to satisfy the requirements of the General Nursing Council. Employment for the registered nurse will continue to be as it has been for the unregistered nurse, good, bad, and indifferent. Good for the first ten years after the completion of her training, and from then on declining to uncertainty. Good for those who have pre-eminently the qualities acceptable to patients, for those who have introductions to first-rate institutes and medical men in large practice, good for those at the top of the tree. But these, even among registered nurses, will remain the minority. For the rest employment can never rise above the general level of the public ability to pay fees. With every sympathy for the desire to reduce unlawful competition, we are constrained to recognise that the registered nurse who falls behind in the race will, under the proposed legislation, enjoy no higher opportunities for getting work than she does at present. If her coveted letters lead her to the American resource of attempting to force up fees they will do her an ill service indeed.

Everyone is unanimously in favour of reforms which will ensure that trained nurses have been thoroughly taught their business. But to confer permanent State recognition on nurses who comply with certain conditions, seems in the eyes of many nursing authorities a doubtful course, in the interests both of nurses and of the public.

